# Reduced Cycle Spinning Method for the Undecimated Wavelet Transform

**DOI:** 10.3390/s19122777

**Published:** 2019-06-20

**Authors:** Miguel A. Rodriguez-Hernandez

**Affiliations:** Instituto ITACA, Universitat Politècnica de València, Camino de Vera, s/n, Valencia 46022, Spain; marodrig@upvnet.upv.es; Tel.: +34-963-877-000 (ext. 79309)

**Keywords:** cycle spinning, undecimated wavelet transform, computational cost, denoising, ultrasonic

## Abstract

The Undecimated Wavelet Transform is commonly used for signal processing due to its advantages over other wavelet techniques, but it is limited for some applications because of its computational cost. One of the methods utilized for the implementation of the Undecimated Wavelet Transform is the one known as Cycle Spinning. This paper introduces an alternative Cycle Spinning implementation method that divides the computational cost by a factor close to 2. This work develops the mathematical background of the proposed method, shows the block diagrams for its implementation and validates the method by applying it to the denoising of ultrasonic signals. The evaluation of the denoising results shows that the new method produces similar denoising qualities than other Cycle Spinning implementations, with a reduced computational cost.

## 1. Introduction

The wavelet transforms are commonly used to analyse non-stationary signals and, as a result, there is a wide bibliography both generalist [1,2,3,4] and applied [5,6,7]. The wavelet term is assigned to a set of time frequency transforms that depending on the selection of some parameters produces more specific definitions of wavelet transforms. The most utilized wavelet is the Discrete Wavelet Transform (DWT), which has the disadvantage of not being shift-invariant. The Undecimated Wavelet Transform (UWT) was designed to overcome the lack of the DWT time variance [8]. There are several implementations of the UWT, for instance the à trous algorithm [8], but this work deals with the technique called Cycle Spinning (CS) [9].

The CS method has been used in the processing of different kinds of images: astronomical images [10], synthetic aperture radar images [11], medical images for diagnosis [12] and for estimation of fluid flows [13], or the classic image of Lena [14]. However, CS has been also used for the extraction of signal characteristics from electrical discharges [15], for denoising in electromagnetic signals from geophysical explorations [16] or for denoising of ultrasonic signals used both for nondestructive evaluations [17,18] and for medical diagnosis [19,20].

The CS algorithm has some advantages over other wavelet implementations. The main advantage is that the final processed signal is obtained by the combination of different shifted versions of the processed signal. This characteristic produces a high redundancy of the processed information and allows different points of view, which can suggest different solutions, for instance the best basis selection in wavelet denoising problems. The redundancy of the processed information is intrinsic to the UWT, but the CS UWT technique is more redundant than other UWT techniques. For instance, the à trous algorithm generates (J+1) wavelet coefficients per sample whereas the CS algorithm generates $2^{J}$ (being J the maximum decomposition level of the UWT analysis) [18].

The UWT CS analysis is based on performing numerous DWTs to circular shifted versions of the signal to process, generating the CS coefficients. After the wavelet coefficients processing, the UWT CS synthesis is carried out performing several inverse DWTs (iDWT), resulting in a set of recovered signals that must be combined to obtain the final processed signal. The limitation of the CS method is the high number of DWTs and iDWTs to perform, and the reduction of the CS computational cost has been treated in several works. In References [21,22] it was demonstrated that the CS coefficients, obtained performing DWTs with maximum decomposition level *J*, were periodic every $2^{J}$ shifts. Recently in Reference [18] the complete formulation of the CS coefficients was done and other periodicities of the CS coefficients were shown. These periodicities allowed the proposal of some alternatives to reduce the computational cost in the CS method. One of them was the Partial Cycling Spinning (PCS) method [17,19] that performs the CS method using only a subset of the circular shifted versions of the signal to process, reducing the number of DWTs and iDWTs.

**Figure 1:**
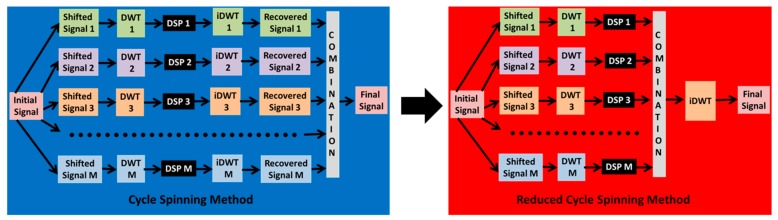
Cycle Spinning method and the proposed Reduced Cycle Spinning method.

The present work introduces an improvement to the CS UWT implementation by removing all the iDWTs except one, reducing the computational cost. The improvement is obtained doing a combination of the wavelet coefficients, after the Digital Signal Processing (DSP) tap and prior to the iDWT tap. In this way, the combination is performed over the wavelet coefficients instead of over the recovered signals, reducing the number of iDWTs to one. Figure 1 shows the schemes of the standard CS method and the proposed Reduced Cycle Spinning (RCS) method.

This work develops the mathematical formulation of the new RCS method, studies its advantages and limitations, evaluates different options for the wavelet coefficients combination and shows experimental results which validate the proposed method. The experimental validation is done by comparing the RCS method with the CS and the PCS methods applied to the denoising of ultrasonic signals. This paper is written in four parts: Section 2 resumes the CS method; Section 3 introduces the new RCS method; Section 4 describes the simulations of an ultrasonic denoising problem and Section 5 explains the conclusions.

## 2. Undecimated Wavelet Transform and Cycle Spinning

The Discrete Wavelet Transform (DWT) is defined by:

(1) $$DWT_{x\left(k\right)}\left(j,n\right)=\overset{k=\infty }{\underset{k=-\infty }{\sum }}x\left(k\right)\frac{1}{\sqrt{2^{j}}}\psi \left(\frac{k}{2^{j}}-n\right)\;\;\;j,n\;\in Z$$

where x(k) is the discrete signal, ψ(k) the mother wavelet, n is the discrete time and j is the scale level.

The popularity of the DWT is in part due to its easy implementation with iterative filter banks using the Mallat algorithm [23]. In the Mallat algorithm the input signal passes through two filters (a low pass filter and a high pass filter) and the outputs of the filters are decimated. The output of the high pass filter is formed by the wavelet coefficients and the output of the low pass filter is the input of the filtering Level 2, which uses the same filter bank as Level 1. Repeating this filtering-decimating process, the wavelet coefficients are obtained for all the decomposition levels.

**Figure 2:**
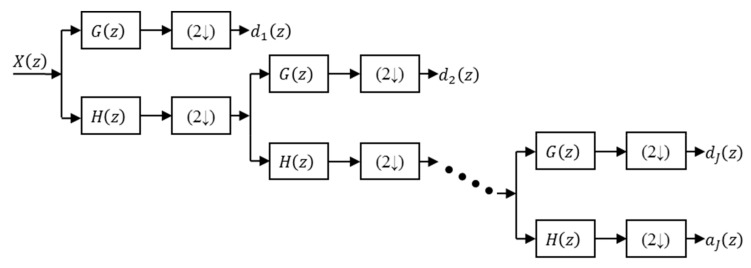
DWT Mallat analysis algorithm with *J* levels.

Figure 2 shows the Mallat DWT analysis algorithm. The input X(z) is the z-transform of x(k), G(z) and H(z) are the transfer functions of the low pass and high pass filters, the blocks with (2↓) represent the decimation by 2, $d_{j}\left(z\right)$ are the wavelet coefficients at the decomposition level j and $a_{J}\left(z\right)$ are the coefficients from the output of low pass filter of level J.

The number of wavelet coefficients obtained by the Mallat algorithm is coincident with the number of samples of the input signal x(k), *K* samples. The expressions of the DWT coefficients are [3]:

(2) $$\begin{array}{c} d_{j}\left(z\right)=X\left(z\right){\left[H\left(z\right)\left(2↓\right)\right]}^{j-1}\left[G\left(z\right)\left(2↓\right)\right]\\ a_{J}\left(z\right)=X\left(z\right){\left[H\left(z\right)\left(2↓\right)\right]}^{J} \end{array}$$

The synthesis of the signal from the wavelet coefficients can be obtained using another filter bank, where two new low and high filters, $\tilde{G}\left(z\right)$ and $\tilde{H}\left(z\right)$ (obtained from G(z) and H(z)), are utilized and where the decimator is replaced by an interpolator. The expression of the recovered signal is:

(3) $$\tilde{X}\left(z\right)=a_{J}\left(z\right){\left[\left(2↑\right)\tilde{H}\left(z\right)\right]}^{J}+\overset{J}{\underset{j=1}{\sum }}d_{j}\left(z\right)\left(2↑\right)\tilde{G}\left(z\right){\left[\left(2↑\right)\tilde{H}\left(z\right)\right]}^{j-1}$$

The DWT is not a shift-invariant transform because the wavelet coefficients of level j are calculated with periods of $2^{j}$ samples [8]. To overcome this problem was developed the Undecimated Wavelet Transform (UWT) that is an invariant-shift transform [8].

(4) $$UWT_{x\left(k\right)}\left(j,n\right)=\overset{k=\infty }{\underset{k=-\infty }{\sum }}x\left(k\right)\frac{1}{\sqrt{2^{j}}}\psi \left(\frac{k-n}{2^{j}}\right)\;\;\;j,n\;\in Z$$

There are two widely used algorithms for the implementation of the UWT: the à trous algorithm [8] and the CS method [9]; the relationship among them was studied in Reference [18]. 

The CS algorithm is based on the DWTs of several circular shifted versions of the input signal x(k). Therefore, it is necessary to calculate a DWT for each shifted version of the input signal. The shifted versions of the input signal, $x_{m}\left(k\right),$ are obtained using the shift operator $Sh_{m}\left[\;\right]$ [18].

(5) $$x_{m}\left(k\right)=Sh_{m}\left[x\left(k\right)\right]=x({\left(k+m\right)}_{\mathrm{mod}\;K})\;\;\;\;\;\mathrm{being}\;{\left(k+m\right)}_{\mathrm{mod}\;K}=\{\begin{array}{c} \left(k+m+K\right)\;\;\;\mathrm{if}\;\left(k+m\right)<0\\ \left(k+m\right)\;\;\;\;\;\mathrm{if}\;0\leq \left(k+m\right)<K\\ \left(k+m-K\right)\;\;\mathrm{if}\;\left(k+m\right)\geq K \end{array}$$

with 0≤k≤K−1 and 0≤m≤M−1, being m the shift and M≤K. $M=2^{J}$ because the CS UWT coefficients are periodic every $2^{J}$ shifts [21,22], thus if M is increased the wavelet coefficient obtained are periodically repeated.

In the z-transform domain the shift operator $Sh_{m}\left[\;\right]$ is transformed to $S_{m}\left[\;\right]\;\;$ and the shifted signals can be expressed as:

(6) $$X_{m}\left(z\right)=TZ\{Sh_{m}\left[x\left(k\right)\right]{\}=S}_{m}\left[X\left(z\right)\right]=\mathrm{Remainder}\left[\frac{X\left(z\right).z^{m}}{z^{-K}}\right]$$

**Figure 3:**
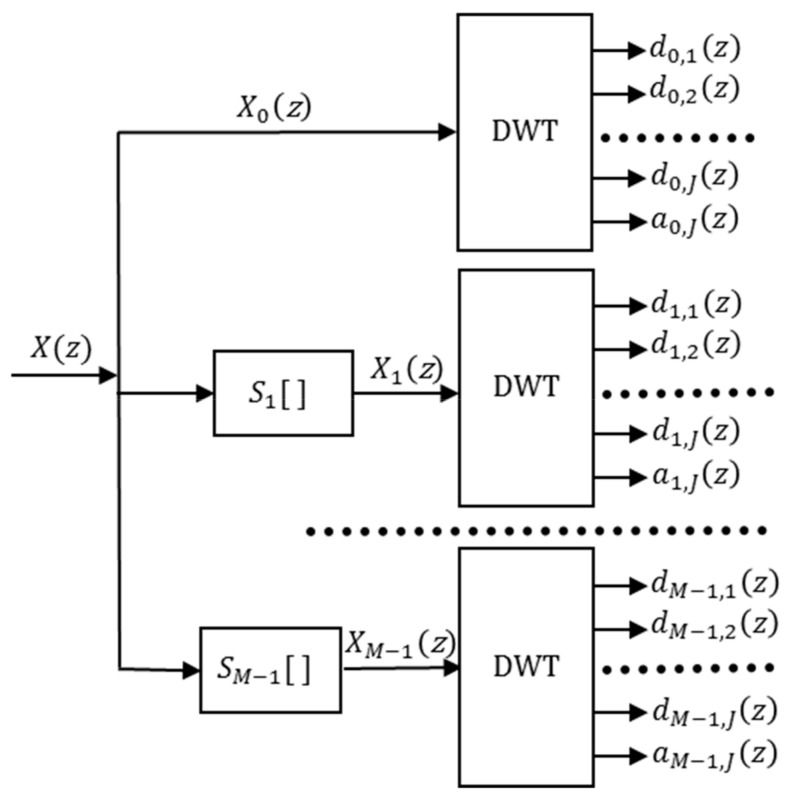
Block diagram of the CS analysis with *M* shifts.

Figure 3 shows the UWT analysis scheme using CS with maximum decomposition level J. The output of the CS analysis is composed of the wavelet coefficients of M DWTs, so the total number of coefficients is $MK=2^{J}K$. The expressions of the CS wavelet coefficients are:

(7) $$\begin{array}{c} d_{m,j}\left(z\right)=X_{m}\left(z\right){\left[H\left(z\right)\left(2↓\right)\right]}^{j-1}{\left[G\left(z\right)\left(2↓\right)\right]}^{}\\ a_{m,J}\left(z\right)=X_{m}\left(z\right){\left[H\left(z\right)\left(2↓\right)\right]}^{J} \end{array}$$

with 0≤m≤M−1 and 1≤j≤J.

**Figure 4:**
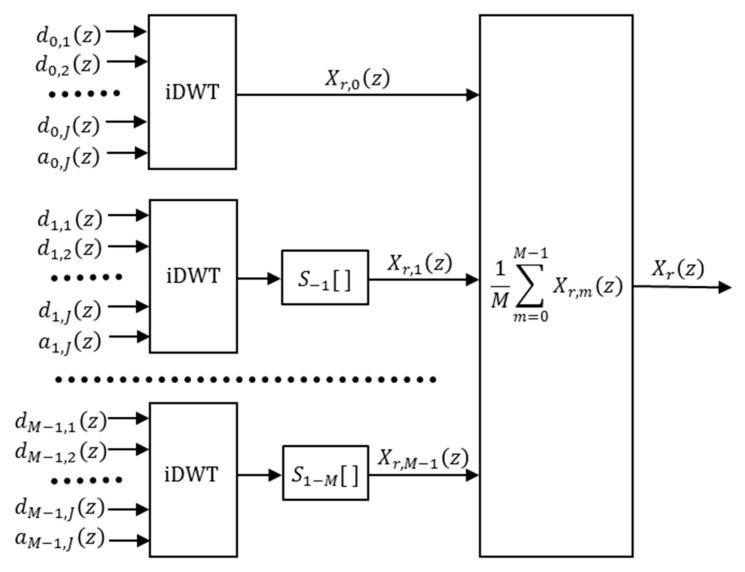
Block diagram of the CS synthesis with *M* shifts.

The synthesis of the recovered signal $X_{r}\left(z\right)$ from the CS wavelet coefficients is carried out by means of several parallel iDWT’s processes, followed by the inverse shifts that compensate the ones performed during the analysis process (see Figure 4). In this way, if the wavelet coefficients have not been modified, the signals recovered for each shift, $X_{r,m}\left(z\right)$, are equal to the original signal X(z). In fact, with the CS method, M processed versions $X_{r,m}\left(z\right)$ of the original signal X(z) are obtained. 

Using the Equation (5), $X_{r,m}\left(z\right)$ can be written as:

(8) $$X_{r,m}\left(z\right)=S_{-m}\left[a_{m,J}\left(z\right){\left[\left(2↑\right)\tilde{H}\left(z\right)\right]}^{J}+\overset{J}{\underset{j=1}{\sum }}d_{m,j}\left(z\right)\left(2↑\right)\tilde{G}\left(z\right){\left[\left(2↑\right)\tilde{H}\left(z\right)\right]}^{j-1}\right]$$

Finally, the recovered signal $X_{r}\left(z\right)$ is obtained by combining the different signals $X_{r,m}\left(z\right)$ using the mean. The Figure 4 show the complete diagram block of the CS synthesis process.

(9) $$X_{r}\left(z\right)=\frac{1}{M}\overset{M-1}{\underset{m=0}{\sum }}X_{r,m}\left(z\right)$$

An alternative to reduce the number of shifts $M=2^{J}$ is the PCS method. The PCS method introduced in References [17,18,19] is an approximation of the CS method with a reduced number of shifts, $M<2^{J}$. The PCS method selects a subset of the shifts and performs the UWT in a similar way to the CS method. The quality of the results obtained with the PCS method depends on the selected subset of shifts, but with an adequate selection of the shifts the quality of the processed signal is maintained. The PCS method can obtain good results with M≅J, reducing the number of shifts from $2^{J}$ to J, and obtaining a computational cost reduction by a factor of approximately $2^{J}/J$. The PCS method optimizes the CS method maintaining the same block diagrams both in analysis and synthesis, but reducing the number of shifts ($M<2^{J}$); the RCS method presented in this work optimizes the CS method using a new synthesis block diagram with only one iDWT.

## 3. Reduced Cycling Spinning (RCS) Method.

The CS algorithm needs a high number of DWTs and iDWTs, one DWT and one iDWT for each signal shift. The number of operations performed in each DWT or iDWT is approximately 2CK, with C being a constant factor that depends on the filter length and K the number of samples of the input signal x(k). Therefore, for the implementation of the CS method with M shifts the number of needed operations is approximately 4CKM, 2CKM for the analysis tap and 2CKM for the synthesis tap. If all the possible shifts are performed, M=K, the number of operations would be approximately $4CK^{2}$. Different methods based on the selection of a limited number of shifts have been proposed to reduce the order of the CS operations from $O\left(K^{2}\right)$ to $O\left(K\log_{2}\left(K\right)\right)$ [10,17,19]. The proposed RCS method eliminates all iDWTs except one, reducing the operations in the synthesis from 2CKM to 2CK. The RCS method maintains the CS analysis tap and proposes a new synthesis process, implying approximately 2CK(M+1) operations. Therefore, the RCS method reduces the number of operations from 4CKM to 2CK(M+1), very close to the half value. In addition, the RCS method can be combined with other approximated CS methods as the ones proposed in References [10,17,19] increasing the computational cost reduction.

In the RCS synthesis the combination of the shifted wavelet coefficients is performed prior to the iDWT and implies some difficulties. The CS synthesis only needs to perform one inverse shift for each shift performed in the analysis block, but the RCS synthesis needs to perform several inverse shifts for each shift performed in the analysis block because the shifts associated to the wavelet coefficients vary depending on the decomposition levels due to decimators. A shift m in the initial signal X(z) generates a shift of m/2 in the coefficients $d_{1}\left(z\right)$, a shift of $m/2^{2}$ in the coefficients $d_{2}\left(z\right)$, and in general a shift of $m/2^{j}$ in the coefficients $d_{j}\left(z\right)$. In this way, to compensate for the effects of a shift m over the wavelet coefficients it is necessary to perform an inverse shift $S_{\left(\frac{-m}{2^{1}}\right)}\left[\;\right]$ for scale 1, an inverse shift $S_{\left(\frac{-m}{2^{2}}\right)}\left[\;\right]$ for scale 2, and in general an inverse shift $S_{\left(\frac{-m}{2^{j}}\right)}\left[\;\right]$ for scale j.

(10) $$\begin{array}{c} d_{m,j}^{s}\left(z\right)=S_{\left(\frac{-m}{2^{j}}\right)}\left[d_{m,j}^{}\left(z\right)\right]\\ a_{m,J}^{s}\left(z\right)=S_{\left(\frac{-m}{2^{J}}\right)}\left[a_{m,J}^{}\left(z\right)\right] \end{array}$$

On the other hand, the ratio $m/2^{j}$ is not an integer in all cases and it is necessary to approximate the fractional shifts to an integer in order to perform the inverse coefficients shifts. The approximation of $m/2^{j}$ to an integer is done by rounding, which is an easy operation and minimizes the maximum rounding shift error.

(11) $$\begin{array}{c} {\tilde{d}}_{m,j}^{s}\left(z\right)=S_{\mathrm{round}\left(\frac{-m}{2^{j}}\right)}\left[d_{m,j}^{}\left(z\right)\right]\\ {\tilde{a}}_{m,J}^{s}\left(z\right)=S_{\mathrm{round}\left(\frac{-m}{2^{J}}\right)}\left[a_{m,J}^{}\left(z\right)\right] \end{array}$$

The rounding shift error assumed doing the approximation of Equation (11) is:

(12) $$\varepsilon_{m,j}=\frac{m}{2^{j}}-\mathrm{round}\left(\frac{m}{2^{j}}\right)=\frac{c}{2^{j}}$$

where c is an integer which value depends on m and varies between $-2^{j-1}+1$ and $2^{j-1}$.

The module of the maximum rounding shift error is bounded to 1/2 for all the scales. 

The rounding shift error $\varepsilon_{m,j}$ generates another error in the wavelet coefficients proportional to the $\varepsilon_{m,j}$ value. The approximated shifted coefficients, ${\tilde{d}}_{m,j}^{s}(z$) and ${\tilde{a}}_{m,J}^{s}\left(z\right)$, can be expressed as the exact shifted coefficients, $d_{m,j}^{s}\left(z\right)$ and $a_{m,J}^{s}\left(z\right)$, plus an error:

(13) $$\begin{array}{c} {\tilde{d}}_{m,j}^{s}\left(z\right)=d_{m,j}^{s}\left(z\right)+e_{d}(\varepsilon_{m,j})\\ {\tilde{a}}_{m,J}^{s}\left(z\right)=a_{m,J}^{s}\left(z\right)+e_{a}(\varepsilon_{m,J}) \end{array}$$

being $e_{d}(\varepsilon_{m,j})$ and $e_{a}(\varepsilon_{m,J})$ the errors of the wavelet coefficients.

The combination of the shifted coefficients is performed with the mean. Other alternatives for the combination (maximum and minimum) are evaluated in the experimental part, but results confirm the mean as the best option. The exact combined coefficients, $d_{j}\left(z\right)$ and $a_{J}\left(z\right)$, that would be obtained using the exact and unavailable shifted coefficients, $d_{m,j}^{s}(z$) and $a_{m,J}^{s}\left(z\right)$, have the expressions:

(14) $$\begin{array}{c} d_{j}\left(z\right)=\frac{1}{M}\overset{M-1}{\underset{m=0}{\sum }}d_{m,j}^{s}\left(z\right)\\ a_{J}\left(z\right)=\frac{1}{M}\overset{M-1}{\underset{m=0}{\sum }}a_{m,J}^{s}\left(z\right) \end{array}$$

However, in practice the proposed RCS algorithm use the approximated shifted coefficients, ${\tilde{d}}_{m,j}^{s}(z$) and ${\tilde{a}}_{m,J}^{s}\left(z\right)$, for combination and the resulting approximated combined wavelet coefficients ${\tilde{d}}_{j}\left(z\right)$ and ${\tilde{a}}_{J}\left(z\right)$ are:

(15) $$\begin{array}{c} {\tilde{d}}_{j}\left(z\right)=\frac{1}{M}\overset{M-1}{\underset{m=0}{\sum }}{\tilde{d}}_{m,j}^{s}\left(z\right)=\frac{1}{M}\;\overset{M-1}{\underset{m=0}{\sum }}\left[d_{m,j}^{s}\left(z\right)+e_{d}(\varepsilon_{m,j})\right]=d_{j}\left(z\right)+\frac{1}{M}\;\overset{M-1}{\underset{m=0}{\sum }}e_{d}(\varepsilon_{m,j})=d_{j}\left(z\right)+{\bar{e}}_{d}(\varepsilon_{m,j})\\ {\tilde{a}}_{J}\left(z\right)=\frac{1}{M}\overset{M-1}{\underset{m=0}{\sum }}{\tilde{a}}_{m,J}^{s}\left(z\right)=\frac{1}{M}\;\overset{M-1}{\underset{m=0}{\sum }}\left[a_{m,J}^{s}\left(z\right)+e_{a}(\varepsilon_{m,J})\right]=a_{j}\left(z\right)+\frac{1}{M}\;\overset{M-1}{\underset{m=0}{\sum }}e_{a}(\varepsilon_{m,J})=a_{j}\left(z\right)+\;{\bar{e}}_{a}(\varepsilon_{m,J}) \end{array}$$

The approximated coefficients ${\tilde{d}}_{j}\left(z\right)$ and ${\tilde{a}}_{J}\left(z\right)$ are the exact coefficients $d_{j}\left(z\right)$ and $a_{J}\left(z\right)$ plus the mean errors of the shifted wavelet coefficients, ${\bar{e}}_{d}(\varepsilon_{m,j})$ and ${\bar{e}}_{a}(\varepsilon_{m,j})$. These mean errors depend on the coefficients values distribution and it is not possible to calculate them in a general way. However, ${\bar{e}}_{d}(\varepsilon_{m,j})$ and ${\bar{e}}_{a}(\varepsilon_{m,J})$ also depend on the mean of the rounding shift error the value of which is:

(16) $${\bar{\varepsilon }}_{m,j}=\frac{1}{2^{j}}\overset{2^{j-1}}{\underset{c=-2^{j-1}+1}{\sum }}\frac{c}{2^{j}}=\frac{1}{2^{j}}\frac{2^{j-1}}{2^{j}}=\frac{1}{2^{j+1}}$$

The mean rounding shift error, ${\bar{\varepsilon }}_{m,j}$, has a low fixed value for each scale and decreases in an exponential way with the scale resulting negligible for many scales. As ${\bar{e}}_{d}(\varepsilon_{m,j})$ and ${\bar{e}}_{a}(\varepsilon_{m,J})$ depend on ${\bar{\varepsilon }}_{m,j}$, it is possible to conclude that ${\bar{e}}_{d}(\varepsilon_{m,j})$ and ${\bar{e}}_{a}(\varepsilon_{m,J})$ also have low values, decrease in an exponential way with the scale and in many scales are negligible. In this way, the high scales errors affect a large number of samples of the recovered signals but they are very low, whereas the low scales errors could be more important but affect only a small number of samples of the recovered signals.

Finally, the signal ${\tilde{X}}_{r}\left(z\right)$ is recovered from the wavelet coefficients ${\tilde{d}}_{j}\left(z\right)$ and ${\tilde{a}}_{J}\left(z\right)$ with a unique iDWT.

(17) $${\tilde{X}}_{r}\left(z\right)={\tilde{a}}_{J}\left(z\right){\left[\left(2↑\right)\tilde{H}\left(z\right)\right]}^{J}+\overset{J}{\underset{j=1}{\sum }}{\tilde{d}}_{j}\left(z\right)\left(2↑\right)\tilde{G}\left(z\right){\left[\left(2↑\right)\tilde{H}\left(z\right)\right]}^{j-1}\;=\;\left[a_{J}\left(z\right)+{\bar{e}}_{a}(\varepsilon_{m,J})\right]{\left[\left(2↑\right)\tilde{H}\left(z\right)\right]}^{J}+\overset{J}{\underset{j=1}{\sum }}\left[d_{j}\left(z\right)+{\bar{e}}_{d}(\varepsilon_{m,j})\right]\left(2↑\right)\tilde{G}\left(z\right){\left[\left(2↑\right)\tilde{H}\left(z\right)\right]}^{j-1}\;=X_{r}\left(z\right)+{\bar{e}}_{a}(\varepsilon_{m,J}){\left[\left(2↑\right)\tilde{H}\left(z\right)\right]}^{J}+\overset{J}{\underset{j=1}{\sum }}{\bar{e}}_{d}(\varepsilon_{m,j})\left(2↑\right)\tilde{G}\left(z\right){\left[\left(2↑\right)\tilde{H}\left(z\right)\right]}^{j-1}=X_{r}\left(z\right)+\delta \left(z\right)$$

The approximated recovered signal ${\tilde{X}}_{r}\left(z\right)$ is the exact recovered signal, $X_{r}\left(z\right),$ plus an error. The error in the recovered signal is:

(18) $$\delta \left(z\right)={\bar{e}}_{a}(\varepsilon_{m,J}){\left[\left(2↑\right)\tilde{H}\left(z\right)\right]}^{J}+\overset{J}{\underset{j=1}{\sum }}{\bar{e}}_{d}(\varepsilon_{m,j})\left(2↑\right)\tilde{G}\left(z\right){\left[\left(2↑\right)\tilde{H}\left(z\right)\right]}^{j-1}$$

The error δ(z) depends on ${\bar{e}}_{d}(\varepsilon_{m,j})$ and ${\bar{e}}_{a}(\varepsilon_{m,J})$, ${\bar{e}}_{d}(\varepsilon_{m,j})$ and ${\bar{e}}_{a}(\varepsilon_{m,J})$ depend on ${\bar{\varepsilon }}_{m,j}$ that has low values; so the error δ(z) will have low values.

**Figure 5:**
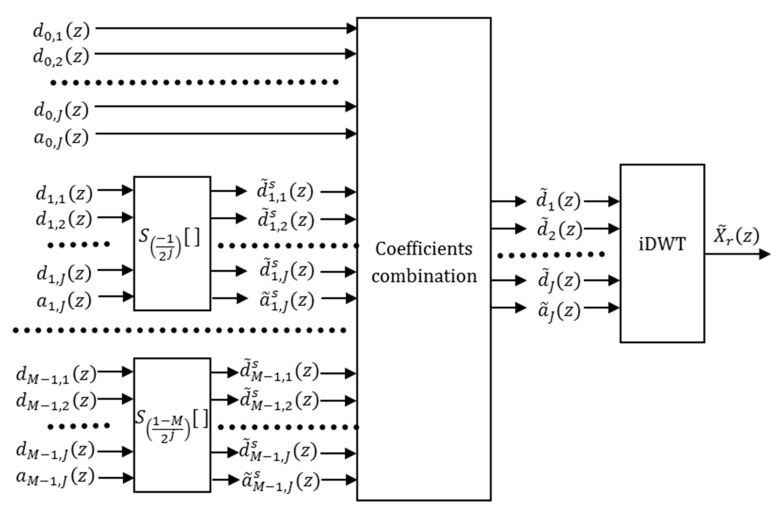
Block diagram of the RCS synthesis with *M* shifts.

Figure 5 displays the block diagram of the complete RCS synthesis using the approximated wavelet coefficients.

## 4. Experimental Results

The goodness of the proposed RCS method is confirmed by applying RCS to a denoising problem of ultrasonic signals. In this way, in the following subsections an introduction to the wavelet signal denoising problem is done, to later describe the ultrasonic experiments carried out and show the results obtained after performing the experiments.

### 4.1. Signal Denoising Using CS

The denoising of signal using wavelets was introduced by Donoho and Johnstone in different works [24,25,26]. The denoising processing with wavelets is divided into three basic steps: the first is the wavelet coefficients calculation of the signal to denoise, the second is the denoising of the wavelet coefficients by means of thresholding, and the third is the synthesis of the signal from the processed coefficients. 

There are different types of thresholds [27,28], but the three most commonly used in wavelet denoising are: the Minimax threshold [24], the Universal threshold [25,26] and the SURE threshold [25].

The Universal threshold is defined by:

(19) $$T_{U}=\frac{\mathrm{median}\left(\left|d_{m,j}\right|\right)}{0,6745}\sqrt{2\ln N_{j}}$$

being $N_{j}$ is the number of $d_{m,j}$ coefficients at level j.

The Minimax threshold is:

(20) $$T_{Mm}=\frac{\mathrm{median}\left(\left|d_{m,j}\right|\right)}{0,6745}\lambda^{*}\left(N_{j}\right)$$

where λ*($N_{j}$) is defined in Table 1 of Reference [24].

A risk function is used for the SURE threshold that in the case of soft thresholding [29] follows the expression:

(21) $$SURE\left(T;X\right)=N-2\;.\#\left\{i:\left|X_{i}\right|\leq T\right\}+\overset{N}{\underset{i=1}{\sum }}{\left[\min\left(\left|X_{i}\right|,T\right)\right]}^{2}$$

being $\#\left\{i:\left|X_{i}\right|\leq T\right\}$ the cardinal of $\left\{i:\left|X_{i}\right|\leq T\right\}$.

The SURE threshold is the value that minimizes:

(22) $$T_{S}=\arg\min_{T\geq 0}\left\{SURE\left(T;\frac{d_{m,j}}{\mathrm{median}\left(\left|d_{m,j}\right|\right)/0,6745}\right)\right\}$$

### 4.2. Experimental Setup

Several sets of synthetic ultrasonic traces were generated to the experimental verification of the RCS method. Each synthetic trace was generated by the insertion of a real ultrasonic echo acquired from a 1 MHz frequency transducer in a simulated ultrasonic grain noise register. The coloured ultrasonic grain noise registers, N(f), came from a noise simulator based on the model proposed in Reference [30]:

(23) $$N\left(f\right)=\left[N_{1}\left(f\right)f^{2}H_{t}\left(f\right)\right]\exp\left(\alpha_{0}f^{4}\right)+N_{2}\left(f\right)$$

where $H_{t}\left(f\right)$ is the frequency response of the ultrasonic transducer, $N_{1}\left(f\right)$ models the structure of the material, $\alpha_{0}$ is the attenuation and $N_{2}\left(f\right)$ is the additive white Gaussian noise associated to the measuring equipment. 

The registers corresponding to the ultrasonic echo and the grain noise were added with different weights to generate several sets of traces with different signal to noise ratios (SNR). The SNR was used as the quality parameter for both the initial signal and the processed signals. SNR was defined by References [28,30,31,32]:

(24) $$\mathrm{SNR}=\frac{\mathrm{peak}\;\mathrm{value}\;\mathrm{in}\;\mathrm{target}\;\mathrm{zone}}{\mathrm{standard}\;\mathrm{deviation}\;\mathrm{of}\;\mathrm{trace}}$$

Seven sets of 1000 traces with different SNR were generated. The length of each trace was *K* = 4096 samples and the sampling frequency was 64 MHz, thus the length of the simulated registers was 64 microseconds. The value of the attenuation parameter of noise was $\alpha_{0}=1.8\;\times \;10^{-26}.$

### 4.3. Experimental Results 

The simulations presented in this paper were done using MATLAB over a Windows PC with Intel Core i7-4790k CPU and 16 MB of RAM. Denoising processing was performed for the seven sets of noisy ultrasonic registers with the CS, PCS and RCS method, using CS and PCS as references. For all methods, Daubechies 6 [2] was selected as mother wavelet due to the previous experience with this kind of signals [17,18,19,20], J = 7 was the maximum decomposition level, the filters border treatment was zero padding and soft thresholding was used with threshold values calculated independently for each decomposition level [27]. RCS was applied in combination with the PCS method to obtain a greater reduction of the computational cost. The number of shifts used for both the RCS and the PCS methods was M = 8, the selected shifts values for the two methods were m∈{3, 14, 51, 61,88, 97, 104, 108}. Three evaluations were carried out varying the threshold: Minimax, SURE and Universal. Additionally, in the RCS method three alternatives for the combination of the wavelet coefficients were studied: mean, maximum and minimum.

Two types of results were obtained: numerical and graphical. The numerical results are displayed in Table 1, Table 2 and Table 3 where the mean SNR values of the seven sets of 1000 ultrasonic traces processed with different methods are listed. Table 1, Table 2 and Table 3 contain six columns and the differences among tables are due to the threshold utilized in the denoising. The first column values are common for the three tables and correspond to the initial SNR of the ultrasonic traces. Columns 2 and 3 show the reference results obtained using the CS and PCS methods and the three last columns list the SNR values using the RCS method with the three combination choices: mean, minimum and maximum. 

Table 1, Table 2 and Table 3 show that the SNR results of the CS, PCS and RCS methods are similar. Although the RCS method was proposed using the mean for the coefficients combination, the SNR results using the minimum and the maximum are close to the mean results and in some cases they are even slightly better. However, the mean choice generates registers in which the non-eliminated noise peaks are lower than in the minimum and maximum cases, even lower than in the CS and PCS cases.

**Figure 6:**
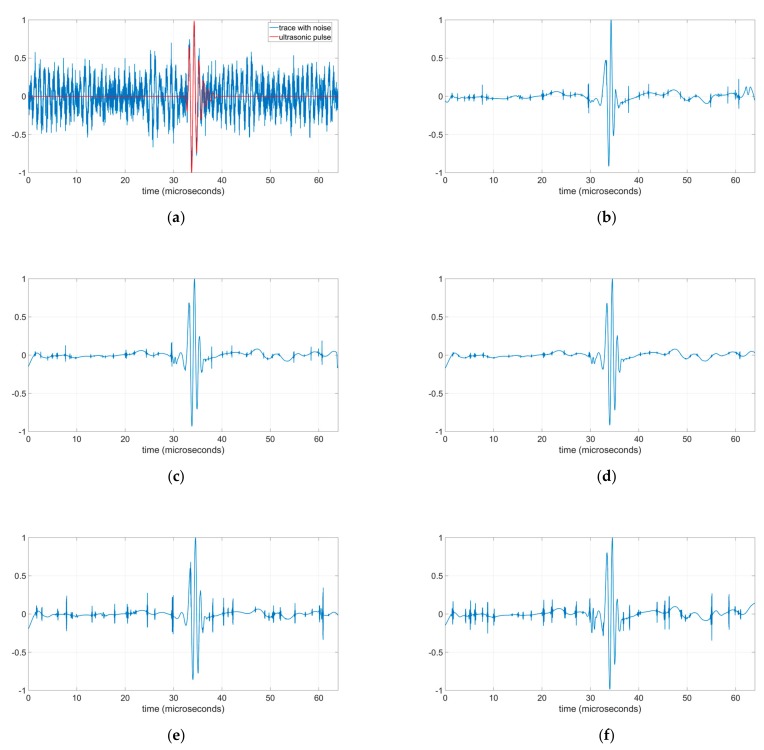
Ultrasonic traces after denoising with different methods using a soft Minimax threshold: (**a**) Ultrasonic pulse and initial trace with noise, SNR = 4.24 and MSE = 0.0413; (**b**) Denoised trace with CS method, SNR = 9.08 and MSE = 0.0072; (**c**) Denoised trace with PCS method using eight shifts, SNR = 8.18 and MSE = 0.0066; (**d**) Denoised trace with RCS method using eight shifts and mean to combine coefficients, SNR = 8.26 and MSE = 0.0062; (**e**) Denoised trace with RCS method using eight shifts and minimum to combine coefficients, SNR = 8.25 and MSE = 0.0086; (**f**) Denoised trace with RCS method using eight shifts and maximum to combine coefficients, SNR = 7.95 and MSE = 0.0092.

Figure 6b–e show the normalized graphical results of the denoised ultrasonic trace with noise of Figure 6a with different wavelet methods using the Minimax threshold. The processed signals of Figure 6c–f were processed using the same eight shifts and have an SNR very close to 8.2. However, it can be clearly observed that Figure 6d, obtained with RCS and mean, shows lower noise peaks values outside the area of the recovered pulse than in Figure 6e,f. Furthermore, these non-eliminated noise peaks are also lower in Figure 6d than in Figure 6b,c, obtained using CS and PCS respectively. This noise peaks reduction effect is another improvement observed in the denoising RCS method with coefficients combination using the mean. The cause of the noise peak reduction has not been determined with detail and probably it is due to the averaging of the wavelet coefficients; this is an open problem for a future research. 

An additional quality parameter was calculated to evaluate the similitude between the denoised signals of Figure 6b–e and the ultrasonic pulse without noise of Figure 6a, the Minimum Square Error (MSE) defined as:

(25) $$\mathrm{MSE}=\frac{1}{K}\overset{K-1}{\underset{k=0}{\sum }}{\left({\tilde{x}}_{r}\left(k\right)-P\left(k\right)\right)}^{2}$$

where ${\tilde{x}}_{r}\left(k\right)$ is the recovered denoised signal and P(k) the ultrasonic pulse without noise.

The MSE of the initial trace with noise (Figure 6a) is 0.0413 and it is reduced after the denoising by all the methods. The best MSE after denoising is obtained, again, by the RCS method with mean (MSE = 0.0062), even this value is slightly better than MSE PCS value (MSE = 0.0066) and MSE CS value (MSE = 0.0072). The RCS methods with minimum and maximum have MSE values of 0.0086 and 0.0092 respectively.

**Figure 7:**
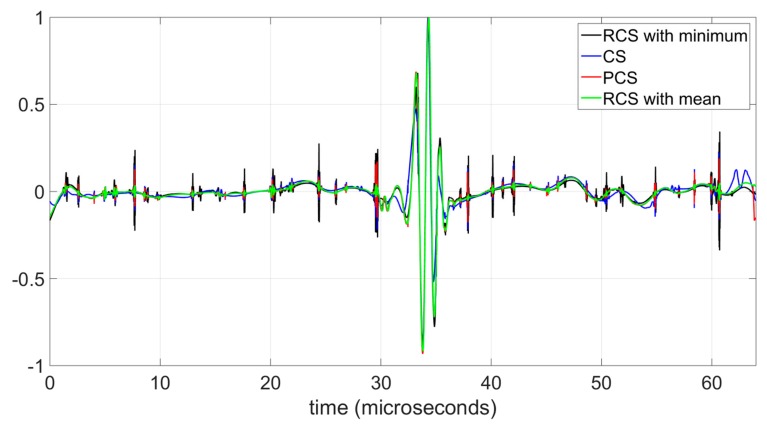
Comparative of denoised ultrasonic traces with four methods using a soft Minimax threshold.

Figure 7 shows the joint comparison of the denoised ultrasonic registers of Figure 6b–e. The individual curves are shown in Figure 6. In Figure 7 the curves are superimposed to compare the reduction of the residual noise and the perfect synchronization of the recovered signals. To evaluate the residual noise in Figure 7, the curves are ordered according to the noise level; the curve with the greatest residual noise (RCS with minimum) is represented at the deepest level whereas the curve with the lowest residual noise (RCS with mean) is represented at the most superficial level. In Figure 7 the green line corresponds to the RCS method with mean and shows the lowest values in the noise peaks. The highest values of the noise peaks correspond to RCS method using minimum (black line), whereas the registers associate to CS and PCS have intermediate values. Additionally, Figure 7 shows the perfect time synchronization achieved in the recovered signals using the RCS method. The perfect time synchronization and the low values of the residual noise confirm the goodness of the RCS method.

### 4.4. Error Evaluation.

Another experiment was designed to evaluate the influence of the rounding shift error over the recovered signal. In this experiment only the RCS analysis and synthesis of an ultrasonic pulse were performed (without the denoising), and the initial and the recovered pulses were compared. In the RCS synthesis part, the rounding shift error for all the scales was selected equal to 1/2. This error corresponds to the theoretical worse case and it cannot happen in a real problem because there is not a shift that generates rounding shift errors equal to 1/2 for all the scales.

The simulation used Daubechies 6, J = 7 and zero padding as in the previous denoising experiments. To simplify the representation but without loss of generality, the shifts m=0 and m=1 were selected to calculate the RCS analysis wavelet coefficients and to generate the RCS shifted wavelet coefficients with rounding shift errors. In this way, the shifted wavelet coefficients with rounding shift error equal to 1/2 for all the scales were obtained using:

(26) $$\begin{array}{c} {\tilde{d}}_{0,j}^{s}\left(z\right)=d_{0,j}^{s}\left(z\right)+\frac{d_{0,j}^{s}\left(z\right)-d_{1,j}^{s}\left(z\right)}{2}\\ {\tilde{a}}_{0,j}^{s}\left(z\right)=a_{0,j}^{s}\left(z\right)+\frac{a_{0,j}^{s}\left(z\right)-a_{1,j}^{s}\left(z\right)}{2} \end{array}$$

Note that a rounding shift error equal to 1/2 implies the error of the shifted wavelet coefficients correspond to a half of the difference between the wavelet coefficients of two consecutive shifts, 0 and 1 in the present case.

**Figure 8:**
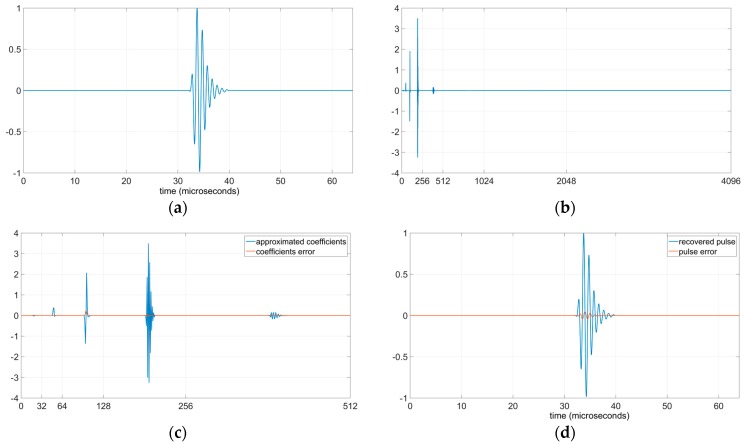
Effects of a rounding shift error equal to 1/2 in all the scales. (**a**) Ultrasonic pulse to analyse and to synthetize; (**b**) Wavelet coefficients with m=0; (**c**) Approximated shifted wavelet coefficients (with rounding shift error influence) for scales j=4, 5, 6 and 7, and error of the wavelet coefficients; (**d**) Recovered pulse after RCS analysis and synthesis, and difference between pulses of Figure 8a,d.

Figure 8 shows the initial and the recovered pulses, the wavelet coefficients and the errors committed during the RCS process in both wavelet coefficients and recovered pulse. Figure 8a displays the initial ultrasonic pulse. Figure 8b represents the wavelet coefficients of the ultrasonic pulse, in this figure it can be appreciated that the coefficients are different to 0 only in scales j=4, 5, 6 and 7. Figure 8c shows the scales j=4, 5, 6 and 7 of the approximated shifted wavelet coefficients and the error associated to each approximated shifted wavelet coefficient. In this figure it can be appreciated the low magnitude of the wavelet coefficients error. Figure 8d contains the curves of the recovered pulse and the difference (error) between the initial pulse and the recovered pulse. In this figure it can be observed the low magnitude of the error in the recovered pulse. An MSE of 3.1 × 10^−5^ confirms the low magnitude of the error in the recovered pulse. 

A second experiment was designed to evaluate the errors in the RCS method with real shifts. In this experiment the ultrasonic pulse was analysed and synthetized with the set of shifts used in the denoising experiment, m∈{3, 14, 51, 61,88, 97, 104, 108}. The rounding shift errors $\varepsilon_{m,j}$ associated to each shift m and scale j are listed in Table 4. The mean of the rounding shift errors associated to each scale is in all the scales inferior to the 1/2 value used in the previous error evaluation.

**Figure 9:**
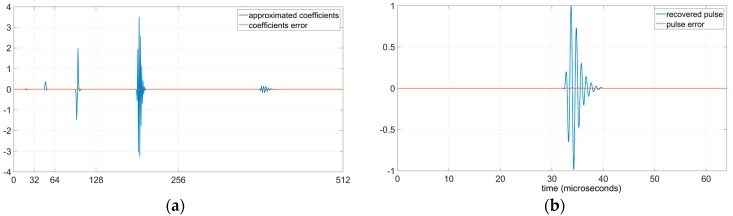
Effects of the rounding shift error with m∈{3, 14, 51, 61,88, 97, 104, 108}. (**a**) Approximated shifted wavelet coefficients for scales j=4, 5, 6 and 7 and error of the wavelet coefficients; (**b**) Recovered pulse after RCS analysis and synthesis, and difference between pulses of Figure 8a and Figure 9b.

Figure 9a displays the approximated shifted wavelet coefficients and its error. As in Figure 8c, they only represent the scales j=4, 5, 6 and 7 with coefficients values different to 0. The magnitude of the coefficients error of Figure 9a is inferior to the magnitude of the coefficients error of Figure 8a. Figure 9b shows the recovered pulse and the error of the recovered pulse. The error of the recovered pulse is also inferior to the error in Figure 8d; the MSE in this case is 1.8 × 10^−6^.

Two experiments have been performed to evaluate the errors in the RCS method. The first experiment simulates the theoretical and non-real case where the rounding shift error is maximum (1/2) for all the scales; the second experiment corresponds to the set of shifts used in the denoising problem. Both experiments confirm that although the error associated to the recovery signal using RCS method depends on each specific signal, the magnitudes of the errors is low compared with the magnitudes of the shifted wavelet coefficients and the recovered signal. Additionally, the MSE values confirm the low magnitude of the errors.

## 5. Conclusions

This paper introduces the RCS method for the CS UWT implementation that reduces the computational cost by a factor close to 2 and is compatible with other optimized methods such as PCS. The proposed RCS method maintains the analysis blocks of the CS method but changes the synthesis tap reducing the number of iDWTs to one. For the iDWT’s reduction in the synthesis tap, it is necessary to perform a combination over the wavelet coefficients, instead of over the recovered signals as in the CS method.

The combination of the wavelet coefficients associated to each shift has implied the proposal of solutions to the difficulties that appeared during development. The main difficulty was due to the fact that the shifts have integer values in the shifted signals, but not in the CS wavelet coefficients because of the decimation process. To solve this problem and calculate the inverse shifts during the synthesis process, the shifts were rounded and a rounding shift error was generated. The rounding shift error was studied and its effects over the recovered signal were estimated. The error in the recovered signal depends on the mean value of the rounding shift error, and the mean value of the rounding shift error has been calculated resulting ${\bar{\varepsilon }}_{m,j}=\frac{1}{2^{j+1}}$. Therefore, the error in the recovered signal is proportional to $\frac{1}{2^{j+1}}$, negligible in most of the samples; the experimental results confirm this hypothesis.

The novelty of the proposed RCS method is the combination of the wavelet coefficients associated with each shift, so different alternatives for the coefficients combination have been studied. Specifically, the paper shows results using three alternatives for the coefficients combination: the mean, the maximum and the minimum. In denoising processing applications, the SNR and MSE values do not show big differences among the coefficients combination methods. 

On the other hand, the graphical results of denoised ultrasonic traces show an interesting effect, the non-eliminated noise peaks are smaller using the RCS method with the mean than using RCS with the minimum or maximum. Additionally, there is a reduction of these noise peaks compared to the CS and PCS methods. The reduction of the non-eliminated noise peaks in RCS denoising must be studied to determinate the causes and to evaluate the effects of applying RCS to other signal processing problems. 

The final conclusion is that this work introduces the RCS method for the CS UWT implementation with half the computational cost of the standard CS method, develops the RCS mathematical formulation, shows the block diagrams of the RCS analysis and synthesis taps, performs an experimental validation applying the RCS method to an ultrasonic denoising problem and evaluates the RCS errors. 

## Figures and Tables

**Table 1:** SNR mean of 1000 ultrasonic traces sets after denoising with different methods using Universal threshold.

Initial	CS	PCS	RCS mean	RCS max.	RCS min.
3.09	0.91	0.92	0.93	0.68	1.24
3.89	2.81	2.67	2.70	1.881	3.46
4.74	6.53	6.39	6.36	5.11	6.65
5.61	8.91	8.88	8.81	8.54	8.74
6.49	9.36	9.28	9.27	9.48	9.40
7.33	9.28	9.29	9.31	9.39	9.50
8.13	9.02	8.92	8.94	9.17	9.18

**Table 2:** SNR mean of 1000 ultrasonic traces sets after denoising with different methods using Minimax threshold.

Initial	CS	PCS	RCS mean	RCS max.	RCS min.
3.09	3.31	3.04	2.83	2.97	3.74
3.89	6.42	6.34	6.28	5.95	6.48
4.74	8.59	8.54	8.50	7.88	8.43
5.61	9.12	8.94	8.94	8.96	9.19
6.49	9.14	8.99	8.97	9.15	9.18
7.33	9.03	8.91	8.92	9.12	9.17
8.13	8.84	8.80	8.80	9.06	9.14

**Table 3:** SNR mean of 1000 ultrasonic traces sets after denoising with different methods using SURE threshold.

Initial	CS	PCS	RCS mean	RCS max.	RCS min.
3.09	3.79	3.72	3.67	3.20	4.05
3.89	6.10	6.05	6.03	5.45	5.95
4.74	7.62	7.61	7.58	6.86	7.42
5.61	8.28	8.26	8.25	7.90	8.29
6.49	8.54	8.50	8.51	8.30	8.53
7.33	8.64	8.56	8.57	8.65	8.74
8.13	8.64	8.61	8.62	8.75	8.79

**Table 4:** Rounding shift error $\varepsilon_{m,j}$ for m∈{3, 14, 51, 61,88, 97, 104, 108} and J=7.

m	*j* = 1	*j* = 2	*j* = 3	*j* = 4	*j* = 5	*j* = 6	*j* = 7
3	1/2	−1/4	3/8	3/16	3/32	3/64	3/128
14	0	2/4	−2/8	−2/16	14/32	14/64	14/128
51	1/2	−1/4	3/8	3/16	−13/32	−13/64	51/128
61	1/2	1/4	−3/8	−3/16	−3/32	−3/64	61/128
88	0	0	0	8/16	−8/32	24/64	−40/128
97	1/2	1/4	1/8	1/16	1/32	−31/64	−31/128
104	0	0	0	8/16	8/32	−24/64	−24/128
108	0	0	4/8	−4/16	12/32	−20/64	−20/128
mean	1/4	2/16	3/32	9/64	7/128	25/256	7/512
